# Position statement of the Brazilian Palliative Care Academy on withdrawing and withholding life-sustaining interventions in the context of palliative care

**DOI:** 10.62675/2965-2774.20240021-en

**Published:** 2024-08-28

**Authors:** Edison Iglesias de Oliveira Vidal, Sabrina Correa da Costa Ribeiro, Maria Júlia Kovacs, Luciano Máximo da Silva, Daniele Pompei Sacardo, Simone Brasil de Oliveira Iglesias, Josimário João´da Silva, Cinara Carneiro Neves, Diego Lima Ribeiro, Fernanda Gomes Lopes

**Affiliations:** 1 Universidade Estadual Paulista "Júlio de Mesquita Filho" Faculdade de Medicina de Botucatu Internal Medicine Department Botucatu SP Brazil Geriatrics Discipline, Internal Medicine Department, Faculdade de Medicina de Botucatu, Universidade Estadual Paulista "Júlio de Mesquita Filho" - Botucatu (SP), Brazil.; 2 Universidade Federal do Ceará Internal Medicine Department Intensive Care Discipline Fortaleza CE Brazil Intensive Care Discipline, Internal Medicine Department, Universidade Federal do Ceará - Fortaleza (CE), Brazil.; 3 Universidade de São Paulo Psicology Institute São Paulo SP Brazil Psicology Institute, Universidade de São Paulo - São Paulo (SP), Brazil.; 4 Hospital Santo Antônio Palliative Care Service Blumenau SC Brazil Palliative Care Service, Hospital Santo Antônio - Blumenau (SC), Brazil.; 5 Universidade Estadual de Campinas Faculdade de Ciências Médicas Public Health Department Campinas SP Brazil Bioetics Discipline, Public Health Department, Faculdade de Ciências Médicas, Universidade Estadual de Campinas - Campinas (SP), Brazil.; 6 Universidade Federal de São Paulo Escola Paulista de Medicina Hospital São Paulo São Paulo SP Brazil Pediatric Intensive Care Unit, Hospital São Paulo, Escola Paulista de Medicina, Universidade Federal de São Paulo - São Paulo (SP), Brazil.; 7 Universidade Federal de Pernambuco Medical Sciences Center Recife PE Brazsil Medical Sciences Center, Universidade Federal de Pernambuco - Recife (PE), Brazsil.; 8 Hospital Infantil Albert Sabin Fortaleza CE Brazil Hospital Infantil Albert Sabin - Fortaleza (CE), Brazil.; 9 Instituto Escutha Fortaleza CE Brazil Instituto Escutha - Fortaleza (CE), Brazil.

**Keywords:** Palliative care, Withholding treatment, Clinical decision-making, Bioethics, Ethics, medical, Consensus

## Abstract

The issue of withrawing and withholding life-sustaining interventions is an important source of controversy among healthcare professionals caring for patients with serious illnesses. Misguided decisions, both in terms of the introduction/maintenance and the withdrawal/withholding of these measures, represent a source of avoidable suffering for patients, their loved ones, and healthcare professionals. This document represents the position statement of the Bioethics Committee of the Brazilian Palliative Care Academy on this issue and establishes seven principles to guide, from a bioethical perspective, the approach to situations related to this topic in the context of palliative care in Brazil. The position statement establishes the equivalence between the withdrawal and withholding of life-sustaining interventions and the inadequacy related to initiating or maintaining such measures in contexts where they are in disagreement with the values and care goals defined together with patients and their families. Additionally, the position statement distinguishes strictly futile treatments from potentially inappropriate treatments and elucidates their critical implications for the appropriateness of the medical decision-making process in this context. Finally, we address the issue of conscientious objection and its limits, determine that the ethical commitment to the relief of suffering should not be influenced by the decision to employ or not employ life-sustaining interventions and warn against the use of language that causes patients/families to believe that only one of the available options related to the use or nonuse of these interventions will enable the relief of suffering.

*I found her in complete despair. Nothing could calm her. No word, phrase or gesture was capable of soothing or dispelling such poignant pain. "Why was she intubated? That was not her wish!" "I no longer recognize her among tubes, probes, devices." "Her body was already dead, now her soul, her dignity, her history are dying." When the pain allowed for a moment of silence, she then told us that her mother was afflicted with advanced lung disease and severe heart disease, with no possibility of treatment that would cure or prolong life with quality. Her mother's wish was to die at home, in her room, her bed; for her life had been in her home, and so should her death. When she was admitted, she was taken to the emergency room, separated from her daughter; there was no dialog, no questions, except for the triage, in which mother and daughter were able to say that intubation and mechanical ventilation would not fit in her treatment. They explained that they had already developed advance directives with the attending physicians and, given the absence of a disease-modifying treatment, comfort would be best. And comfort was not having pain. At that moment, the daughter, immersed in her memories, lonely, desolate, isolated, looking down the empty hallway, still finds the strength to say: "We have no choice. We have no voice, and when we try to speak, we are not heard. It wasn't what she wanted. This pain that I live now is the pain of impotence. Not even now, so close to death, can we have dignity. Not even now can we have respect, consideration. We have no voice. And my tears have dried up, because even crying is useless."*

Report from the daughter of a patient

## INTRODUCTION

The issue of withdrawing and withholding life-sustaining interventions is a major source of controversy among healthcare professionals caring for patients with serious and life-threatening illnesses.^(1-3)^ Misguided decisions, both in terms of the introduction/maintenance and the withdrawing/withholding of these measures, represent a source of avoidable suffering for patients, their loved ones, and healthcare professionals. This document represents the position statement of the Bioethics Committee of the Brazilian Palliative Care Academy (ANCP - *Academia Nacional de Cuidados Paliativos*) on this issue and establishes principles to guide, from a bioethical perspective, the approach to situations related to this topic in the context of palliative care in Brazil. The text is organized into two main sections, namely, a preamble, in which we present the arguments that justify the elements that make up the position statement, and the position statement itself.

## METHODS

Similar to other national and international position statements of scientific societies on topics related to ethical issues,^(4-9)^ an iterative process was used to arrive at a consensus. An interdisciplinary committee composed of specialists in palliative care, intensive care (adult and pediatric), emergency medicine, internal medicine, geriatrics, psychology, and bioethics reviewed the literature on the subject and held online meetings over approximately 1 year and 6 months to discuss the elements that should guide the present position statement on the withdrawing and withholding of life-sustaining interventions. One of the authors wrote the first version of the manuscript, which was iteratively revised and improved through multiple virtual meetings. Finally, the content of the position statement was evaluated and approved by the ANCP board.

### Preamble

In the last century, important changes occurred in the way individuals living in Western societies experience the end of life. Several technical advances in medicine have made it possible for clinical situations previously incompatible with life to become manageable, and the very distinction between "saving a life" and "prolonging death" has become less clear in many situations.^(10)^ Thus, concerns related to the end of life have shifted from merely the occurrence of a premature death to also including the possibility of a delayed death, occurring slowly through the undue prolongation of life by advances in medical technology, in situations of dependence contrary to the individual values of the patients.^(11,12)^

Until the mid-20^th^ century, medical ethics prioritized the principles of beneficence and nonmaleficence, and the autonomy of patients was not even considered relevant.^(13)^ Consequently, it was natural for healthcare professionals to adopt a paternalistic posture, in which the main guide of the treatment plan was what the physician judged to be ‘the best’ for his or her patient. Thus, physicians were allowed to implement treatments and perform procedures without even obtaining the consent of the patients who would be subjected to them. This type of attitude was based on the premise that these professionals were able to discern the conduct that would best meet the interests and needs of patients without having to ask them about their perspectives on such issues.^(14)^

It was only in the second half of the 20^th^ century, through court decisions in the United States, which granted people the right to consent or refuse medical treatments, that the principle of autonomy began to be incorporated into medical ethics.^(13)^ Since then, it has been expected that patients’ informed consent be obtained before performing medical procedures, and the right of patients to refuse treatments proposed by healthcare teams has also been validated.^(15)^

Importantly, the strengthening of the principle of autonomy was listed as the main contribution of the 2009 edition of the Brazilian Code of Medical Ethics,^(16)^ which highlights the long period for the establishment of this principle in the regulations that govern medical practice in Brazil. In fact, it was only in 2011 that the National Health Council (CNS - *Conselho Nacional de Saúde*) published the Charter of Patients’ Rights, ratifying their right to exercise informed consent (or refusal) regarding medical procedures.^(17)^

In 2012, the Federal Medical Council (CFM - *Conselho Federal de Medicina*) took a new step toward strengthening the autonomy of patients through its Resolution No. 1995, in which it defined Advance Directives as the "set of wishes, previously and expressly manifested by the patient, about the care and treatment that they want to receive or not when they are unable to express their will freely and autonomously".^(18)^ This resolution affirms the duty of physicians to take these directives into account when making decisions about the care and treatment of patients who are unable to express their will independently.

By strengthening the principle of autonomy, the practice of healthcare professionals began a trajectory toward a more thoughtful posture focused on meeting the needs of the patient. This stance emerged in a global context in which the behavior of healthcare professionals became guided not only by Principlism Ethics but also by other bioethical perspectives.^(19-21)^ The main practical consequence of this bioethical movement was the strengthening of shared decision-making as the main method for establishing a therapeutic care plan.^(5,22)^ Being aware of these aspects of the evolution of medical ethics throughout history is essential to understand the bioethical basis for withdrawing and withholding artificial life-sustaining treatments.

The first case to receive authorization from a court of justice for the withdrawal of ventilatory life support occurred in the United States in 1976. Young Karen Ann Quinlan, 22 years old, was under mechanical ventilation (MV) and in a persistent vegetative state for approximately 9 months as a result of an overdose.^(13)^ The adoptive father and legal guardian of the patient requested that the MV of his daughter be withdrawn because he understood that this measure was at odds with her values and those of her family. However, the hospital refused to comply with the family's request, and most medical societies at the time also opposed the withdrawal of artificial life support.^(23)^ The case of the patient reached the Supreme Court of the State of New Jersey; the plaintiffs argued that if, by some miracle, the patient became lucid and aware of her condition of irreversible disability, she would not want to be kept on MV indefinitely. The court ruled that the patient had the right to refuse the maintenance of such treatment and that, as she was unable to make her own decisions, her guardian should decide on her behalf. Additionally, to support the hospital and its medical staff, as well as ensure their legal protection, the court determined that, if the physicians truly believed that she was in a persistent vegetative state and, with the agreement of the hospital's Ethics Committee, ventilatory support of the patient could be withdrawn, without the doctors or the hospital being held civilly or criminally responsible for her death. The patient's ventilatory support was withdrawn in 1976, and, against the expectations of the healthcare team, the patient lived until 1985, when she died of meningitis and pneumonia.

During the trial of this case, the court refuted the arguments of the medical team that there was a difference between withholding and withdrawing artificial life-sustaining treatments for patients with no prospect of cure/recovery.^(24)^

On the one hand, it is important to recognize that the argument of physicians at that time—and of many healthcare professionals today—about the withdrawal of a treatment being morally different from its withholding, is based on the tendency of people in their day-to-day lives to attribute greater responsibility and, therefore, potential for punishment, to situations in which there was an action than when there was an omission.^(25)^ However, such a perception represents an illusion within the scope of medical practice, in which it is understood that the decision not to implement a certain treatment is also an action. For example, all medical interventions (including life-sustaining therapies such as hydration and nutrition) need to be actively prescribed periodically to be maintained in the patient's care plan. Therefore, it is essential to recognize that the distinction between withdrawing and withholding a treatment is not consistent with a logical evaluation of common situations related to health care at the end of life.

The following thought experiment illustrates this paradigm shift.^(26)^ Imagine that an elderly woman with advanced dementia who lives in a nursing home starts showing signs of respiratory distress and is taken to the emergency department. The patient's husband arrives at the emergency department shortly after the ambulance and explains to the attending physician that the patient's care goals, which had even been documented through an advance directive, involved only comfort measures and that she should not be subjected to invasive treatments such as MV or cardiopulmonary resuscitation, which she and her family viewed as a form of torture in that specific context. The patient then receives medication to relieve dyspnea and, showing significant improvement, is transferred to the palliative care ward. In a second version of the story, the patient's husband is delayed by traffic and, when he arrives at the emergency department, finds his wife already intubated and on MV. Now, would it be ethical to keep this patient on MV when there is clear evidence that such treatment violates her values and care preferences, expressed through her representative and a specific document? Would it be appropriate to keep the patient on MV against her will, in a situation that the patient and her family perceived as equivalent to torture, just because her husband was trapped in traffic? This example aims to ratify that, from a bioethical perspective, there is no real distinction between withholding and withdrawing a treatment because the same arguments that justify the withholding of an intervention also justify its withdrawal and the final consequences of both are similar.

Despite the arguments that, from a bioethical and even rational standpoint, the withdrawal and withholding of life-sustaining treatments are equivalent, it is known that, from the point of view of emotions, it is common for healthcare professionals and laypeople to have more conflicting feelings related to the withdrawal of such treatments than to their nonimplementation. In this sense, it is important to recognize the right of healthcare professionals to conscientious objection,^(27)^ which aims to protect the integrity of professionals when they have individual convictions that morally conflict with the duties required by professional practice. The precept of conscientious objection allows healthcare professionals to abstain from participating in procedures that, although legally recognized, are in dissonance with their individual moral values.

It is, therefore, a mechanism to resolve conflict between the responsibility of the professional and their individual rights.^(28)^ However, as the exercise of conscientious objection by healthcare professionals impacts a patient's right to access procedures, it is essential that mechanisms are implemented to minimize and, ideally, eliminate its effects on the patient's rights. Therefore, there is broad international recognition regarding the limits of conscientious objection, which involve situations in which conscientious objection causes suffering, damage, or death to the patient or in which there is an absence of another healthcare professional to assume responsibility for performing, withholding or even withdrawing a given procedure in a timely manner.^(9,29,30)^

One of the most enlightening international position statements on the issue of conscientious objection declares that it should be used as a shield to protect the moral integrity of healthcare professionals, not as a sword to impose the values of professionals on patients.^(9)^ In other words, the purpose of the precept of conscientious objection is to remove a healthcare professional from a specific context of care, not to deny a patient access to a given procedure, which, of course, may even involve the withdrawal of a life-sustaining intervention. The authors of that position statement strongly assert that the conscientious objection of an individual healthcare professional should not be sufficient to determine the type of health care that a patient will or will not receive.

Interestingly, one of the most relevant aspects of that consensus document is almost ‘hidden’ in its online supplement, in which six clinical cases involving conscientious objection are discussed.^(9)^ One of these cases corresponds to that of a 45-year-old man with intracranial hemorrhage secondary to an aneurysm and consistent information supporting his families’ request to withdraw MV. However, the weekend intensive care unit (ICU) attending physician objected to this on conscientious grounds. Revealingly, the position defended by the committee of the American Thoracic Society (ATS) was that, in that case, if it was not possible for another professional to assume responsibility for the removal of MV in a timely manner, the harm caused to the patient and his family owing to the improper maintenance of that procedure outweighed the right to conscientious objection of the attending physician. Therefore, the healthcare professional should comply with such a request, despite their personal convictions, in the name of their professional commitment to protect their patients and families from suffering.

Notably, in 2006, the CFM started to allow physicians either to withhold or to withdraw life-sustaining treatments for patients in the terminal phase of serious and incurable disease, provided that the wishes of the patient or his or her legal representative are respected.^(31)^ Notably, the two main justifications described by the CFM to support this resolution lie in Articles 1 and 5 of the Brazilian Federal Constitution, which establish the principle of human dignity as one of the foundations of our country and prohibit the submission of any person to torture or inhuman or degrading treatment. In other words, CFM Resolution 1,805/2006 allowed physicians to withdraw or withhold life-sustaining treatments to preserve the dignity of people because, in terminal circumstances and depending on the values and preferences of care of the patient, life-support measures could constitute inhumane treatment and be equivalent to a form of torture. An extremely relevant implication of this CFM resolution is that it facilitates the performance of therapeutic trials involving a variety of treatments, including life-support measures, something that is often essential to achieve clarity about their real consequences and impact on patients’ quality of life. If life-sustaining treatments, once initiated, could not be discontinued, the very concept of a therapeutic trial would become unrealistic, significantly complicating the decision-making process.

For example, consider the following situation: a middle-aged patient with a severe respiratory infection caused by the severe acute respiratory syndrome coronavirus 2 (SARS-CoV-2) or another virus develops acute respiratory failure but does not consent to MV due to fear of becoming "dependent on machines for the rest of his life" or "of dying connected to a machine in the ICU". The assurance from the physician responsible for their care that there is a concrete chance of surviving the acute infection phase and recovering with the introduction of MV and admission to the ICU, and that, if the outcome of the patient's condition is unfavorable, MV can be withdrawn so that the patient does not die connected to the ventilator in the ICU, can play a decisive role in the patient's decision to accept a *trial* involving both ICU admission and MV.

Importantly, a fundamental step in addressing issues related to the withdrawal or withholding of life-sustaining treatments involves the use of language in which the key concepts have been clearly defined. In fact, it is an essential prerequisite to prevent communication and decision-making errors. In this sense, it is essential to understand that the concept of "life-sustaining treatments" refers to interventions aimed at maintaining the functioning of different organs and physiological systems with the purpose of prolonging life, but which, by themselves, are incapable of reversing the individual's underlying disease.^(32)^ These measures include cardiopulmonary resuscitation, MV, renal replacement therapy, the use of vasoactive drugs, artificial nutrition and hydration, and the use of antimicrobials. Other concepts fundamental to clinical and bioethical deliberations involving these issues are those of strictly futile interventions and potentially inappropriate treatments, as described below.

According to an important consensus document jointly published in 2015 by five international intensive care societies, only medical interventions in which there is no possibility of achieving their physiological goal (for example, increasing oxygen saturation and restoring spontaneous circulation) should be considered *strictly futile*.^(4)^ Examples of strictly futile interventions involve the use of antimicrobials against pathogens known to be resistant to them and the performance of cardiopulmonary resuscitation in patients whose cardiac arrest is due to refractory multiple organ dysfunction or to massive hemorrhage known to be irreversible.^(25)^ Potentially inappropriate treatments were defined as those in which their physiological goal has some possibility of being achieved but whose ethical basis is questionable.^(33)^ The distinction between strictly futile treatments and potentially inappropriate treatments is fundamental because strictly futile treatments are always a technical and bioethical error and should not be implemented or maintained under any circumstances. Potentially inappropriate treatments, on the other hand, require a shared and more complex deliberation process that involves the identification of the cultural values of the patients and their families, the value that these individuals attribute to certain states of functionality, and the probabilities of the success of the interventions.^(5)^ This need for deliberation arises because the concept of potentially inappropriate treatment, as opposed to strict futility, does not rely exclusively on technical judgment but involves moral values that often vary among individuals. Of course, this does not mean that communication concerning requests from patients or families for strictly futile treatments does not require a dialog marked by sensitivity and cultural humility.^(34)^ Table 1S in the online Supplementary Material provides an example of appropriate communication involving this type of situation. Tables 1 and 2 present examples of strictly futile and potentially inappropriate treatments, as well as the reasons that justify their classification.

**Table 1:** Examples of strictly futile treatments and rationale for determining this judgment

Examples of strictly futile treatments	Rationale
Initiating cardiopulmonary resuscitation procedures in a patient with cardiac arrest due to refractory hypotension from septic shock despite the best possible circulatory support	In these situations, cardiopulmonary resuscitation procedures will not be able to address the problem of refractory hypotension that caused cardiac arrest, and the physiological goal of reestablishing spontaneous circulation is not feasible
Initiating cardiopulmonary resuscitation procedures in a patient with cardiac arrest due to refractory hypoxia despite the best possible ventilatory support	In these situations, cardiopulmonary resuscitation procedures will not be able to address the problem of refractory hypoxia that caused cardiac arrest, and the physiological goal of reestablishing spontaneous circulation is not feasible
Prescribing of antifungal medication for patients with staphylococcal sepsis without clinical or laboratory evidence of fungal infection	There is no pathophysiological basis to justify this treatment and no possibility of the treatment achieving the physiological goal of treating the patient's infection
Cardiopulmonary resuscitation procedures in patients with *rigor* or *livor mortis* in the absence of severe hypothermia as a cause of cardiac arrest	In the absence of severe hypothermia, *rigor* and *livor mortis* are clinical signs of irreversible death and usually indicate that more than 1 hour has elapsed since the interruption of spontaneous circulation, with no possibility of recovery^(35,36)^
Maintaining cardiopulmonary resuscitation procedures for more than 30 minutes for patients with asystole or refractory pulseless electrical activity, who have already received hight-quality resuscitation efforts and whose potentially reversible causes have already been addressed to the extent possible in the given care context	In the absence of new elements indicating that cardiopulmonary resuscitation procedures, which have been extensively attempted and failed to restore spontaneous circulation, could become successful, their continuation alone will not be able to achieve the physiological goal of restoring spontaneous circulation

**Table 2:** Examples of potentially inappropriate treatments and rationale for determining this judgment

Examples of potentially inappropriate treatments	Rationale
Use of tube feeding for patients with advanced dementia	On the one hand, there is evidence suggesting that artificial feeding in patients with advanced dementia does not increase their probability of survival and is associated with a greater likelihood of adverse events, such as pressure sores and use of physical restraints, than comfort feeding.^(7)^ On the other hand, the physiological goal of providing calories and nutrients can be achieved through these interventions. Additionally, it is important to consider that individuals with different cultural perspectives may have distinct frameworks for weighing the risks and benefits of tube feeding
Implementation of mechanical ventilation for patients with metastatic cancer	The presence of metastatic cancer, by itself, does not prevent the physiological goal of mechanical ventilation (e.g., reducing hypoxemia or hypercarbia). In certain contexts, mechanical ventilation may increase the patient's lifespan and enable them to achieve personal goals (e.g., attending the graduation of a grandchild). In other circumstances, the context and culture of the patient may interpret such a procedure as an "undue prolongation of dying"
Use of antibiotics for the treatment of recurrent aspiration pneumonia in a bedridden patient with severe sequelae of stroke	Except in cases of extremely multidrug-resistant germs for which no antimicrobial options are available, in general, there is the possibility that such agents will achieve their physiological goal of eliminating the bacteria responsible for the patient's infection. The appropriateness of continuing to prescribe antibiotics for these infections should take into account the extent to which the patient would like to be kept alive in their current state of health or would consider such a state as a condition worse than death, something closely related to their personal values
Initiating hemodialysis for patients in a chronic vegetative state	The chronic vegetative state alone does not prevent hemodialysis from achieving its physiological goal of purifying the patient's blood of substances that would usually be removed by kidney function, if it were preserved. The central issue here involves a value judgment on the risk/cost benefit ratio of introducing such a procedure for patients with minimal probabilities of regaining consciousness and/or functionality

Unfortunately, it is still common for the lack of knowledge, or misunderstandings of these definitions and their implications for decision-making in the healthcare field to lead to inappropriate and ethically reprehensible conduct, whether in maintaining, withdrawing, or withholding life-support interventions. The case cited in the epigraph of this position statement portrays a situation in which the healthcare team implemented and maintained life-support measures for a patient with serious and incurable diseases when she wanted only a painless death, preferably at home. Had the team recognized that the initiation of invasive MV for that patient was a potentially inappropriate treatment, when the team became aware of the values clearly expressed by the patient and her family, they would have recognized this treatment as definitely inappropriate, and such treatment would not have even been initiated.

Another case recently published in the New England Journal of Medicine described the situation of a middle-aged man with functional dependence due to a neurological disorder associated with difficult-to-control epilepsy, frequent falls and recurrent head trauma.^(37)^ The patient was hospitalized due to aspiration pneumonia, and despite having recovered from pneumonia with antibiotic therapy, continued to have dysphagia with a high risk of aspiration. The patient's family, which included a speech therapist, believed that a gastrostomy would be too invasive and that there was a reasonable chance that the patient could recover the ability to eat by mouth if temporarily fed through a nasogastric tube, as similar episodes had occurred in the past, and he had recovered, contrary to the expectations of many. According to the point of view of the healthcare team, a nasogastric tube would be a source of suffering for the patient. However, his family did not share this judgment because, in the past, this type of tube had been used for a limited period of time, and the patient had tolerated it well. Despite the family's requests for a trial period of nasogastric tube feeding, the patient was discharged from the hospital with a recommendation for a full oral diet. He developed a new episode of pneumonia, and, under pressure from the healthcare team, his family consented for the patient to be transferred to hospice care, where he died a few days later. This case, like the previous one, illustrates the healthcare team's failure to listen to and consider the values of the patient and his family during the decision-making process. The difference is that, in the second case, a method of artificial life-sustaining treatment—the insertion of the nasogastric tube—should initially have been recognized as potentially inappropriate and, through listening and considering the values of the patient/family, would likely have become appropriate.

Additionally, it is essential to recognize that the withdrawal and withholding of life-sustaining interventions in the context of a terminal illness differ from the concept of euthanasia. The International Association for Hospice and Palliative Care (IAHPC) and the European Association for Palliative Care (EAPC) published position statements on the issue of euthanasia,^(38,39)^ defining it as the act of a physician in administering medication to a patient with the intention of causing death based on a voluntary request made by the patient while they had full decision-making capacity. The EAPC document further argued that the term "passive euthanasia" is inappropriate because it represents an intrinsic contradiction, as euthanasia, as defined above, always corresponds to an action and is, therefore, essentially active.^(39)^ Notably, both associations have vehemently opposed euthanasia, stating that it does not align with the philosophical vision contained in the definition of palliative care^(40,41)^ and, therefore, should not be part of the practice of such care. Both the IAHPC and the EAPC were categorical in asserting that the practice of withdrawing or withholding life-sustaining treatments for patients in the terminal phase of serious and incurable diseases does not correspond to euthanasia. One of the central arguments that differentiates euthanasia from the practice of withdrawing/withholding of life-support interventions lies in the fact that, in the former, the drugs administered are the effective cause of death, whereas in the latter, death is caused by the underlying disease and only ceases to be prolonged through the withdrawal or withholding of treatments.

The legality of withdrawing and withholding life-sustaining treatments is already a reality in several countries, such as the United Kingdom, the Netherlands, Australia, Colombia, Taiwan and the United States.^(32)^ Naturally, the question of whether to regulate these practices in each country is influenced by its culture, values, economy and religious orientation.^(42)^ Additionally, these aspects can change over time and throughout the history of countries, such that the withdrawing and withholding of life-support interventions, which were once prohibited, often become accepted at later times.

Even in places where the practice of the withdrawing and withholding of life-sustaining treatments is regulated by law, the knowledge of healthcare professionals is still limited in this regard, leading to the adoption of strictly futile treatments in the face of critical conditions for patients at the end of life.^(32)^

Importantly, the practice of palliative care extends far beyond the mere issue of withdrawing or withholding life-support measures. Palliative care aims to prevent and alleviate the suffering associated with serious and life-threatening diseases and involves adjusting and often withdrawing interventions and treatments that are causing or prolonging suffering in situations of irreversible illness. We must be careful, however, to ensure that we are defining suffering according to the patient's perception and values and not our own concepts. Unfortunately, the term "dysthanasia", which corresponds to the exaggerated prolongation of death,^(43)^ has been misused, based solely on the values of healthcare professionals to qualify as "exaggerated" any situation that they would consider "not worth living", without an adequate exploration of the patients’ values about suffering and what is worth living from their perspective.

Unfortunately, our country has a significant shortage of professionals and teams specializing in palliative care, with 55% of the existing services concentrated in the Southeast region.^(44)^ Considering this deficit, restricting the practice of therapeutic support adjustment, including withdrawing and withholding of life-support treatments, to locations with access to palliative care specialists would limit the access of patients to the possibility of having their values respected and avoid a process of artificially prolonged death against their personal values. We therefore believe that, although the ideal would be to have a team specialized in palliative care^(45)^ (in person or through telemedicine), the adjustment of the therapeutic plan is part of the primary palliative care skills expected of all physicians, as recently recognized by the update of the national curriculum guidelines for undergraduate medical education in Brazil.^(46)^

Finally, the complex process of shared decision-making on whether to employ life-support measures requires a high degree of self-awareness, not only to avoid imposing, albeit in a veiled way, our cultural perspectives^(5)^ but also so that we do not use language that induces misunderstandings in patients and families.^(47)^ For example, the misuse of the verbs "need" and "require" when describing a certain therapeutic option (for example, "If the oxygen levels continue to fall, he will *need to* be intubated and placed on MV") conveys the false impression that this is the only reasonable alternative. In this example, it would be more appropriate to use language such as "If oxygen levels continue to fall, our options are… and involve such advantages and disadvantages, risks and benefits… and may make sense to some people and not to others, depending on their values and preferences".

Unfortunately, it is not uncommon for patients and families to be confronted by healthcare professionals with the false duality of, on the one hand, prolonging life while increasing the person's suffering or, on the other hand, avoiding life-support measures as a strategy to prevent suffering. This approach is inappropriate from a technical and bioethical perspective because, regardless of the preference to use or not use measures to prolong the life of a person within the existing limits, healthcare professionals must always do their best to reduce the suffering of patients. Ultimately, severe and refractory suffering can be relieved through the use of palliative sedation to unconsciousness, regardless of the decision to use or not use life-support measures.^(48,49)^ Naturally, palliative sedation to unconsciousness is a strategy of last resort for the relief of uncomfortable symptoms that have been appropriately evaluated and treated, but without success.^(50)^

### Position Statement of the Brazilian Academy of Palliative Care

In view of the considerations presented in the preamble, the Bioethics Committee of the Brazilian Academy of Palliative Care issues the following principles:

1. From a bioethical point of view, there is no effective distinction between the withdrawal and withholding of life-sustaining interventions. Decisions about the withdrawal or withholding of this type of treatment should always be made following the principles of shared decision-making and require the agreement of the patient or their legal representative.
2. It is unethical to initiate or maintain life-support measures in contexts where these are at odds with the values and goals of care defined together with patients and their families, for example, in situations viewed by patients and their families as worse than death itself.
3. Strictly futile treatments are those that do not have any possibility of achieving even their physiological goal. By definition, strictly futile treatments are not able to prolong life because they are simply ineffective. Therefore, treatments that are strictly futile should not be initiated or maintained even at the request of the patient or his/her representatives.
4. If the maintenance of a given treatment can prolong the life of a patient but raises ethical concerns, such treatment cannot be considered strictly futile and corresponds, in fact, to a potentially inappropriate treatment. The withdrawal or withholding of potentially inappropriate life-support treatments necessarily requires the agreement on therapeutic goals, taking into account the values and perspectives of the patient.
5. It is important to recognize that healthcare professionals, patients and their loved ones may perceive the withdrawal and withholding of life-support interventions as situations with distinct weights and impacts. In these cases, dialogical approaches should be used to bring clarity to all those involved, with sensitivity and empathy, to raise awareness about the central issue of the appropriateness of any intervention, regardless of whether it has already been initiated, to the goals of care agreed upon by healthcare professionals, patients and their families.
6. It is essential to recognize that conscientious objection by healthcare professionals has limits, which involve situations in which it causes patient suffering, harm or death, or in which there is no other healthcare professional to assume responsibility for performing, withholding, or withdrawing a specific intervention in a timely manner.
7. When discussing with patients/families the question of the adequacy of withdrawing or withholding life-support measures, healthcare professionals should avoid using language that leads them to believe that only one of the available options related to the use or nonuse of life support measures will enable the relief of suffering. The ethical commitment to the relief of suffering should not be influenced in any way by the decision to use or not use life-support interventions.
