# Supplementary material for: Position statement of the Brazilian Palliative Care Academy on withdrawing and withholding life-sustaining interventions in the context of palliative care
Source: Crit Care Sci. 2024 Aug 28;36:e20240021en. doi: 10.62675/2965-2774.20240021-en (PMC11463991; doi:10.62675/2965-2774.20240021-en)
Supplement: Supplementary file 1 [file 2965-2774-ccsci-36-e20240021en-suppl01.pdf]

*Critical Care Science*
https://doi.org/10.62675/2965-2774.20240021-en

SUPPLEMENTARY MATERIAL

# Position statement of the Brazilian Palliative Care Academy on withdrawing and withholding life-sustaining interventions in the context of palliative care

Edison Iglesias de Oliveira Vidal[1], Sabrina Correa da Costa Ribeiro[2], Maria Júlia Kovacs[3], Luciano Máximo da Silva[4], Daniele Pompei Sacardo[5], Simone Brasil de Oliveira Iglesias[6], Josimário João da Silva[7], Cinara Carneiro Neves[8], Diego Lima Ribeiro[5], Fernanda Gomes Lopes[9]

**Table 1S -** Example of a communication strategy when facing requests for strictly futile life-sustaining interventions, according to the concept defined in the main text, for a fictitious case

**Background:** A 70-year-old male patient was admitted to the intensive care unit for 12 days due to septic shock after bone marrow transplantation for multiple myeloma. The patient presents refractory hypotension despite the best possible circulatory support, including a progressive increase in the dose of vasoactive drugs. The team anticipates that he will die in the coming hours and, after communicating this difficult news to the family in the most appropriate way possible, the family requests that the patient undergoes cardiopulmonary resuscitation procedures.

**Patient's son:** — We understand the seriousness of my father's situation, but we believe that only God can decide when it is time for him to die. For God, nothing is impossible. Therefore, if his heart stops beating, we would like you to try to resuscitate him.

**Intensivist:** — I understand your pain, your fear of losing your father, and I admire your unshakable faith in God. I think you agree that God always knows what He does, and that it is difficult for us humans to unravel the mysteries of God's designs. We always wish for the miracle of healing, but sometimes the miracle that God assigns to us is to protect the people we love from suffering, and this is already very important, because no one should go through avoidable suffering...

I believe that God puts healthcare professionals in the path of sick people to help and protect them within the limits of their possibilities... That is why I think this conversation of ours is so important…

In the situation your father finds himself in, unfortunately, the infection is so advanced that, despite our most intensive efforts, we are not managing to keep his blood pressure high enough to keep his body organs working properly... and when the pressure continues to drop, as it is happening, there comes a time when the heart can no longer keep beating.

When that happens, unfortunately, it will not do any good to do those movie things and perform compressions on your father's chest, or give him electric shocks, or anything else, because none of that can make his heart start beating again. If we did that, the only thing we would be doing is risking causing him discomfort, risking breaking some of his ribs, knowing in advance that none of this would work. In addition, with these procedures, we would be preventing you and your family from being by his side in his last moments. These are all reasons that prevent us from, in these circumstances, performing those procedures if your father's heart stops beating.

I also wish that God would offer us the miracle of healing for your father… but we need to be prepared for the possibility that God's miracle for your father may be to allow him to die a death without pain or suffering, alongside the people he loves...



This is an open access article under the CC BY license (https://creativecommons.org/licenses/by/4.0/).
