# Supplementary material for: Position statement of the Brazilian Palliative Care Academy on withdrawing and withholding life-sustaining interventions in the context of palliative care
Source: Crit Care Sci. 2024 Aug 28;36:e20240021en. doi: 10.62675/2965-2774.20240021-en (PMC11463991; doi:10.62675/2965-2774.20240021-en)
Supplement: Supplementary file 2 [file 2965-2774-ccsci-36-e20240021en-pt-suppl01.pdf]

Critical Care Science
https://doi.org/10.62675/2965-2774.20240021-pt

MATERIAL SUPLEMENTAR

Posicionamento da Academia Nacional de Cuidados Paliativos sobre suspensão e não implementação de intervenções de suporte de vida no âmbito dos cuidados paliativos

Edison Iglesias de Oliveira Vidal[1], Sabrina Correa da Costa Ribeiro[2], Maria Júlia Kovacs[3], Luciano Máximo da Silva[4], Daniele Pompei Sacardo[5], Simone Brasil de Oliveira Iglesias[6], Josimário João da Silva[7], Cinara Carneiro Neves[8], Diego Lima Ribeiro[5], Fernanda Gomes Lopes[9]

Tabela 1S - Exemplo de estratégia de comunicação diante da solicitação de intervenção de suporte de vida fútil em senso estrito, conforme conceito definido no texto principal, para um caso fictício

Contexto: Paciente do sexo masculino de 70 anos internado em unidade de terapia intensiva há 12 dias em função de choque séptico após transplante de medula óssea para tratamento de mieloma múltiplo. O paciente apresenta hipotensão refratária a despeito do melhor suporte circulatório possível, incluindo o aumento progressivo da dose de drogas vasoativas. A equipe antecipa que ele irá evoluir para óbito nas próximas horas e, após realizar o processo de comunicação dessa notícia difícil à família da forma mais adequada possível, esta solicita que o paciente seja submetido a procedimentos de ressuscitação cardiopulmonar

Filho do paciente: — Nós entendemos a gravidade da situação do meu pai, mas acreditamos que somente Deus pode decidir quando chegou a hora dele morrer. Para Deus nada é impossível. Por isso, gostaríamos que, se o coração dele parar de bater, vocês tentassem ressuscitá-lo

Intensivista: — Eu compreendo sua dor, seu medo de perder seu pai e admiro sua fé inabalável em Deus. Penso que você concorda que Deus sempre sabe o que faz e que é difícil para nós, humanos, desvendarmos os mistérios dos desígnios de Deus. Nós sempre desejamos o milagre da cura, mas, às vezes, o milagre que Deus designa para a gente é proteger as pessoas que amamos do sofrimento, e isso já é muito importante, pois ninguém deve passar por sofrimentos evitáveis...

Eu acredito que Deus coloca os profissionais de saúde no caminho das pessoas doentes para ajudá-las e protegê-las nos limites de suas possibilidades... Por isso, acho que essa nossa conversa é tão importante.

Na situação em que seu pai se encontra, infelizmente, a infecção está tão avançada que, a despeito dos nossos esforços mais intensivos, não estamos conseguindo manter a pressão do sangue dele alta o suficiente para manter os órgãos do corpo dele funcionando direito... e quando a pressão continua caindo, como está acontecendo, chega uma hora em que o coração não consegue mais continuar batendo.

Quando isso acontecer, infelizmente, não vai adiantar fazer aquelas coisas de filme e realizar compressões no peito do seu pai, ou dar choques nele, ou qualquer outra coisa, pois nada disso poderia fazer o coração dele voltar a bater. Se fizéssemos isso, a única coisa que estaríamos fazendo seria arriscar causar desconforto para ele, correr o risco de quebrar algumas costelas dele, sabendo de antemão que nada disso funcionaria. Além disso, com esses procedimentos, estaríamos impedindo que você e sua família estivessem ao lado dele em seus últimos momentos. Esses todos são motivos que nos impedem de, nessas circunstâncias, se o coração do seu pai parar de bater, realizar aqueles procedimentos.

Eu também gostaria que Deus nos oferecesse o milagre da cura para o seu pai, mas precisamos estar preparados para a possibilidade de que o milagre de Deus para seu pai seja permitir que ele tenha uma morte sem dor ou sofrimento, ao lado das pessoas que ele ama.

This is an open access article under the CC BY license (https://creativecommons.org/licenses/by/4.0/).
