# Supplementary material for: Position statement of the Brazilian Palliative Care Academy on withdrawing and withholding life-sustaining interventions in the context of palliative care
Source: Crit Care Sci. 2024 Aug 28;36:e20240021en. doi: 10.62675/2965-2774.20240021-en (PMC11463991; doi:10.62675/2965-2774.20240021-en)
Supplement: Translation

# Translation: Posicionamento da Academia Nacional de Cuidados Paliativos sobre suspensão e não implementação de intervenções de suporte de vida no âmbito dos cuidados paliativos

**DOI:** 10.62675/2965-2774.20240021-pt

**Authors:** Edison Iglesias de Oliveira Vidal, Sabrina Correa da Costa Ribeiro, Maria Júlia Kovacs, Luciano Máximo da Silva, Daniele Pompei Sacardo, Simone Brasil de Oliveira Iglesias, Josimário João da Silva, Cinara Carneiro Neves, Diego Lima Ribeiro, Fernanda Gomes Lopes

**Affiliations:** 1 Botucatu SP Brasil Disciplina de Geriatria, Departamento de Clínica Médica, Faculdade de Medicina de Botucatu, Universidade Estadual Paulista "Júlio de Mesquita Filho" - Botucatu (SP), Brasil.; 2 Fortaleza CE Brasil Disciplina de Terapia Intensiva, Departamento de Medicina Clínica, Universidade Federal do Ceará - Fortaleza (CE), Brasil.; 3 São Paulo SP Brasil Instituto de Psicologia, Universidade de São Paulo - São Paulo (SP), Brasil.; 4 Blumenau SC Brasil Serviço de Cuidados Paliativos, Hospital Santo Antônio - Blumenau (SC), Brasil.; 5 Campinas SP Brasil Disciplina de Bioética, Departamento de Saúde Coletiva, Faculdade de Ciências Médicas, Universidade Estadual de Campinas - Campinas (SP), Brasil.; 6 São Paulo SP Brasil Unidade de Terapia Intensiva Pediátrica, Hospital São Paulo, Escola Paulista de Medicina, Universidade Federal de São Paulo - São Paulo (SP), Brasil.; 7 Recife PE Brasil Centro de Ciências Médicas, Universidade Federal de Pernambuco - Recife (PE), Brasil.; 8 Fortaleza CE Brasil Hospital Infantil Albert Sabin - Fortaleza (CE), Brasil.; 9 Fortaleza CE Brasil Instituto Escutha - Fortaleza (CE), Brasil.

**Keywords:** Cuidados paliativos, Suspensão de tratamento, Tomada de decisão clínica, Bioética, Ética médica, Consenso

## Abstract

O tema da suspensão e da não implementação de intervenções de suporte de vida constitui importante fonte de controvérsia entre profissionais de saúde que lidam com pacientes com doenças graves. Decisões equivocadas, tanto em termos de introdução/manutenção, como de suspensão/não introdução dessas medidas representam fonte de sofrimento evitável para pacientes, seus entes queridos e profissionais de saúde. O presente documento representa o posicionamento do Comitê de Bioética da Academia Nacional de Cuidados Paliativos sobre essa questão e estabelece sete princípios para guiar, do ponto de vista da bioética, a abordagem de situações relacionadas a tal tema no contexto dos cuidados paliativos no Brasil. O posicionamento estabelece a equivalência entre a retirada e a não implementação de intervenções de suporte de vida e a inadequação relacionada a iniciar ou manter tais medidas em contextos em que elas estejam em desacordo com os valores e os objetivos de cuidado definidos em conjunto com pacientes e seus familiares. Adicionalmente, o posicionamento distingue tratamentos fúteis em senso estrito de tratamentos potencialmente inapropriados e elucida sua implicação crucial para a adequação do processo de tomada de decisão médica nesse contexto. Finalmente, o texto aborda a questão da objeção de consciência e seus limites; determina que o compromisso ético pelo alívio do sofrimento não deve ser influenciado pela decisão por empregar ou não intervenções de suporte de vida e adverte contra o uso de linguagem que induza pacientes/familiares a acreditarem que apenas uma das opções disponíveis relacionadas à utilização ou não dessas medidas possibilitará o alívio do sofrimento.

*A encontrei em completo desespero. Nada a acalmava. Nenhuma palavra, frase ou gesto era capaz de pacificar e afugentar uma dor tão pungente. "Por que ela foi entubada? Não era esse o desejo dela!" "Não a reconheço mais entre tubos, sondas, aparelhos". "Seu corpo já estava morto, agora morrendo está sua alma, sua dignidade, sua história". Quando a dor possibilitou um momento de silêncio, ela então nos disse que sua mãe era acometida por uma doença pulmonar avançada e uma doença cardíaca grave, sem possibilidade de tratamento que curasse ou prolongasse a vida com qualidade. O desejo de sua mãe era que a morte ocorresse em domicílio, no seu quarto, sua cama; pois sua vida havia sido no seu lar, e assim deveria ser sua morte. Ao ser atendida, fora conduzida à sala de urgência, a filha afastada, não houve diálogo, perguntas, à exceção da triagem, em que mãe e filha puderam dizer que intubação e ventilação mecânica não caberiam em seu tratamento. Explicaram que já tinham construído diretivas junto aos médicos assistentes e, diante da ausência de um tratamento modificador, o melhor seria o conforto. E conforto era não ter dor. Naquele momento, a filha, imersa em suas recordações, solitária, desolada, isolada, olhando para o corredor vazio, ainda encontra forças para dizer: "Não temos escolhas. Não temos voz e, quando tentamos falar, não somos ouvidas. Não era o que ela queria. Essa dor que vivo agora é a dor da impotência. Nem agora, tão perto da morte, podemos ter dignidade. Nem agora podemos ter respeito, consideração. Não temos voz. E minhas lágrimas já secaram, porque nem chorar adianta mais".*

Relato da filha de uma paciente

## INTRODUÇÃO

O tema da suspensão e não implementação de intervenções de suporte de vida é importante fonte de controvérsia entre profissionais de saúde que lidam com pacientes com doenças graves e ameaçadoras da vida.^(1-3)^ Decisões equivocadas, tanto em termos de introdução/manutenção, como de suspensão/não introdução dessas medidas representam fonte de sofrimento evitável para pacientes, seus entes queridos e profissionais de saúde. O presente documento representa o posicionamento do Comitê de Bioética da Academia Nacional de Cuidados Paliativos (ANCP) sobre essa questão e estabelece princípios para guiar, do ponto de vista da bioética, a abordagem de situações relacionadas a esse tema no contexto dos cuidados paliativos no Brasil. O texto é organizado em duas sessões principais, quais sejam, um preâmbulo, em que são tecidos os argumentos que justificam os elementos que compõem o posicionamento, e o posicionamento propriamente dito.

## MÉTODOS

De forma semelhante a outros posicionamentos nacionais e internacionais de sociedades científicas sobre temas relacionados a questões éticas,^(4-9)^ foi utilizado um processo iterativo para obtenção de consenso. Um comitê interdisciplinar, composto de especialistas em cuidados paliativos, terapia intensiva (de adultos e pediátrica), medicina de urgência, medicina interna, geriatria, psicologia e bioética, procedeu à revisão da literatura sobre o tema e realizou reuniões *on-line* ao longo de cerca de 1 ano e 6 meses, de modo a debater os elementos que deveriam nortear o presente posicionamento sobre suspensão e não implementação de medidas de suporte de vida. Um dos autores redigiu uma primeira versão do manuscrito, que foi revisada e aprimorada iterativamente, por meio de múltiplos encontros virtuais. Finalmente, o conteúdo do posicionamento foi avaliado e aprovado pela diretoria da ANCP.

### Preâmbulo

O século passado foi palco de mudanças importantes na forma como as sociedades ocidentais vivenciam o fim da vida. Diversos avanços técnicos da medicina possibilitaram que situações clínicas previamente incompatíveis com a vida deixassem de sê-lo, e a própria distinção entre "salvar a vida" e "prolongar a morte" tornou-se menos clara em muitas situações.^(10)^ Assim, a preocupação relacionada ao fim da vida deixou de ser apenas quanto à ocorrência de uma morte precoce e passou a envolver também a possibilidade de uma morte tardia, ocorrendo lentamente, por meio do prolongamento indevido de vida pelos avanços da tecnologia médica, em situações de dependência contrárias aos valores individuais dos pacientes.^(11,12)^

Até meados do século 20, a ética médica priorizava os princípios da beneficência e não maleficência, e a autonomia dos pacientes sequer era considerada algo relevante.^(13)^ Consequentemente, era natural que os profissionais de saúde adotassem uma postura paternalista, em que o principal guia do plano terapêutico era o que o médico julgava ser "o melhor" para seu paciente. Desse modo, era permitido aos médicos implementarem tratamentos e realizarem procedimentos, sem sequer terem obtido antes o consentimento por parte dos pacientes que seriam submetidos a eles. Esse tipo de postura baseava-se na premissa de que esses profissionais eram capazes de discernir a conduta que melhor atenderia aos interesses e às necessidades dos pacientes, sem precisar perguntar a eles sobre suas perspectivas em relação a tais questões.^(14)^

Apenas a partir da segunda metade do século 20, mediante decisões judiciais nos Estados Unidos, que garantiram às pessoas o direito de consentir ou não sobre quais tratamentos de saúde que iriam receber, que o princípio da autonomia passou a ser incorporado à ética médica.^(13)^ A partir de então, passa a se esperar que o consentimento livre e esclarecido dos pacientes seja obtido antes da realização de procedimentos médicos, também sendo validado o direito dos pacientes em recusar tratamentos propostos pelas equipes de saúde.^(15)^

É importante chamar atenção para o fato de que o fortalecimento do princípio da autonomia foi listado como a principal contribuição da edição de 2009 do Código de Ética Médica brasileiro,^(16)^ o que ressalta o longo período para o estabelecimento desse princípio nas normativas que regem a prática médica no Brasil. De fato, apenas em 2011, o Conselho Nacional de Saúde (CNS) publicou a Carta dos Direitos dos Pacientes, ratificando o direito deles de exercer o consentimento (ou a recusa) livre e esclarecida quanto à realização de procedimentos médicos.^(17)^

Em 2012, o Conselho Federal de Medicina (CFM) deu um novo passo no sentido de fortalecer a autonomia do paciente por meio de sua resolução 1995, em que define as Diretivas Antecipadas de Vontade como o "conjunto de desejos, prévia e expressamente manifestados pelo paciente, sobre cuidados e tratamentos que quer, ou não, receber no momento em que estiver incapacitado de expressar, livre e autonomamente, sua vontade".^(18)^ Tal resolução afirma o dever dos médicos em levar em consideração tais diretivas quando da tomada de decisões sobre cuidados e tratamentos de pacientes que se encontrem incapazes de expressar de maneira livre e independente suas vontades.

Ao fortalecer o princípio da autonomia, a prática dos profissionais de saúde iniciava uma trajetória em direção a uma postura mais ponderada e focada em atender as necessidades do paciente. Tal postura surge em um contexto mundial, em que o comportamento dos profissionais de saúde passa a ser pautado não só pela Ética Principialista, mas por outras perspectivas bioéticas.^(19-21)^ A principal consequência prática desse movimento bioético foi o fortalecimento da decisão compartilhada como o principal método de decisão para o estabelecimento de um plano terapêutico de cuidados.^(5,22)^ Tomar ciência desses aspectos da evolução da ética médica ao longo da história é essencial para compreender as bases bioéticas para medidas de não implementação, bem como de suspensão de tratamentos de suporte artificial de vida.

O primeiro caso a receber autorização de uma corte de justiça para a suspensão do suporte ventilatório de vida se deu nos Estados Unidos, em 1976. A jovem Karen Ann Quinlan, então com 22 anos de idade, encontrava-se sob ventilação mecânica (VM), em estado vegetativo persistente, há cerca de 9 meses, em consequência de uma *overdose*.^(13)^ O pai adotivo e guardião legal da paciente solicitou que a VM de sua filha fosse suspensa por compreender que se tratava de uma medida que estava em desacordo com os valores da paciente e de sua família. No entanto, o hospital se negou a atender a solicitação da família, e a maior parte das sociedades médicas daquela época também se opôs à retirada do suporte artificial de vida.^(23)^ O caso da paciente chegou à Suprema Corte do estado de Nova Jersey, a qual argumentou que, se, por milagre, a paciente se tornasse lúcida e ciente de sua condição de incapacidade irreversível, não desejaria ser mantida em VM indefinidamente. A corte determinou que a paciente tinha o direito de recusar a manutenção de tal tratamento e que, como ela se encontrava incapaz de tomar suas próprias decisões, seu guardião deveria decidir em seu nome. Adicionalmente, para dar respaldo ao hospital e sua equipe médica, bem como lhes assegurar proteção legal, a corte determinou que, se os médicos realmente acreditassem que ela se encontrava em estado vegetativo persistente e havendo concordância do Comitê de Ética do hospital, o suporte ventilatório da paciente poderia ser suspenso, sem que os médicos ou o hospital fossem responsabilizados civil ou criminalmente por sua morte. O suporte ventilatório da paciente foi retirado em 1976 e, contra as expectativas da equipe de saúde, a paciente viveu até 1985, quando faleceu de meningite e pneumonia.

Durante o julgamento desse caso, a corte refutou os argumentos da equipe médica de que haveria uma diferença entre a não implementação de tratamentos de suporte artificial de vida para pacientes sem perspectiva de cura/recuperação e a suspensão de tais tratamentos para os mesmos pacientes.^(24)^

Por um lado, é importante reconhecer que o argumento dos médicos daquela época - e de muitos profissionais de saúde dos dias de hoje - sobre a suspensão de um tratamento ser moralmente diferente de sua não implementação baseia-se na tendência das pessoas em seu dia a dia a atribuírem maior responsabilidade e, portanto, potencial para punição a situações em que houve uma ação do que quando houve uma omissão.^(25)^ Entretanto, tal percepção representa uma ilusão no âmbito da prática médica, na qual se entende que a decisão por não implementar determinado tratamento também é uma ação. Por exemplo, toda intervenção médica (incluindo as terapias de suporte de vida, como hidratação e alimentação) precisa ser ativamente prescrita periodicamente para ser mantida no plano terapêutico do paciente. Portanto, é fundamental reconhecer que a distinção entre suspender e não implementar um tratamento não condiz com uma avaliação lógica de situações comuns relacionadas aos cuidados de saúde no fim da vida.

O seguinte experimento de pensamento ilustra essa mudança de paradigma.^(26)^ Imagine que uma idosa portadora de uma demência em fase avançada e que vive em uma instituição de longa permanência de idosos passa a apresentar sinais de desconforto respiratório e é levada ao serviço de pronto-socorro. O marido da paciente chega ao pronto-socorro logo em seguida à ambulância e esclarece ao médico plantonista que os objetivos de cuidado da paciente, os quais inclusive tinham sido documentados por meio de uma Diretiva Antecipada de Vontade, envolviam apenas medidas de conforto e que ela não deveria ser submetida a tratamentos invasivos, como VM ou ressuscitação cardiopulmonar, que ela e sua família entendiam representar uma forma de tortura naquele contexto específico. A paciente, então, recebe medicamentos para aliviar a dispneia e, já com importante melhora dos sinais de desconforto, é encaminhada para a enfermaria de cuidados paliativos. Em uma segunda versão da história, o marido da paciente fica preso no trânsito e, quando chega ao pronto-socorro, encontra sua esposa já entubada e em VM. Ora, seria ético manter essa paciente em VM quando há evidências claras de que tal tratamento fere seus valores e preferências de cuidado, manifestos por meio de seu representante e de um documento específico? Seria adequado manter a paciente em VM contra sua vontade, em uma situação que a paciente e sua família percebiam como equivalente a tortura, apenas por que seu marido ficou preso no trânsito? Esse exemplo tem como objetivo ratificar que, do ponto de vista da bioética, não há uma verdadeira distinção entre não introduzir ou suspender um tratamento, porque os mesmos argumentos que justificam a não implementação de uma medida também justificam sua retirada e as consequências finais de ambas as condutas são semelhantes.

A despeito dos argumentos de que, do ponto de vista da bioética e até mesmo da lógica racional, a suspensão e não implementação de medidas de suporte de vida são equivalentes, sabe-se que, do ponto de vista das emoções, é comum que profissionais de saúde e leigos possuam mais sentimentos conflituosos relacionados à suspensão de tais medidas do que à sua não implementação. Nesse sentido, é importante reconhecer o direito dos profissionais de saúde ao dispositivo da objeção de consciência,^(27)^ que tem como objetivo proteger a integridade dos profissionais, quando possuem convicções individuais que conflitam moralmente com os deveres demandados pela prática profissional. O preceito da objeção de consciência autoriza a não participação dos profissionais de saúde em procedimentos que, embora legalmente reconhecidos, estejam em dissonância com seus valores morais individuais.

Trata-se, portanto, de um mecanismo para solucionar situações de embate entre a responsabilidade do profissional e seu direito individual.^(28)^ No entanto, como o exercício da objeção de consciência dos profissionais de saúde impacta o direito dos pacientes ao acesso a procedimentos, é essencial que sejam implementados mecanismos com o objetivo de minimizar e, idealmente, eliminar seus efeitos sobre os direitos do paciente. Para tanto, há amplo reconhecimento internacional, quanto aos limites da objeção de consciência, os quais envolvem situações em que ela causa sofrimento, danos, ou a morte ao paciente, ou em que haja ausência de outro profissional de saúde para assumir a responsabilidade quanto à realização, não implementação ou mesmo a suspensão de determinado procedimento, em tempo hábil.^(9,29,30)^

Um dos posicionamentos internacionais mais esclarecedores sobre a questão da objeção de consciência afirma que ela deve ser usada como um escudo para proteger a integridade moral dos profissionais de saúde, não como uma espada para impor os valores dos profissionais aos pacientes.^(9)^ Em outras palavras, o objetivo do dispositivo da objeção de consciência é remover um profissional de saúde de um determinado contexto de cuidado, não o de negar o acesso de um paciente a determinado procedimento – que, naturalmente, pode, inclusive, envolver a suspensão de uma intervenção de suporte de vida. Seus autores são contundentes ao afirmarem que a objeção de consciência de um profissional de saúde individualmente não deve ser suficiente para determinar o tipo de cuidados de saúde que um paciente irá ou não receber.

Interessantemente, um dos aspectos mais relevantes daquele documento de consenso encontra-se quase que "escondido" em seu suplemento *on-line*, em que são discutidos seis casos clínicos envolvendo objeção de consciência.^(9)^ Um desses casos corresponde ao de um homem de 45 anos com hemorragia intracraniana secundária a um aneurisma e informações consistentes sustentando o pedido de sua família pela suspensão da VM, no entanto, o plantonista da unidade de terapia intensiva (UTI) no fim de semana relatava objeção de consciência a tal conduta. De forma reveladora, a posição defendida pelo comitê da *American Thoracic Society* (ATS) foi a de que, naquele caso, se não fosse possível que outro profissional assumisse a responsabilidade pela retirada da VM em tempo hábil, o prejuízo causado ao paciente e sua família pela manutenção indevida daquele procedimento superava o direito do plantonista à objeção de consciência, e, por isso, o profissional deveria atender tal pedido a despeito de suas convicções pessoais, em nome de seu compromisso profissional de proteger seus pacientes/familiares do sofrimento.

Cabe ainda ressaltar que, em 2006, o CFM passou a permitir aos médicos tanto a não implementação como a suspensão de tratamentos que prolongassem a vida de pacientes em fase terminal de doença grave e incurável, desde que respeitada a vontade do paciente ou de seu representante legal.^(31)^ Merece destaque que as duas principais justificativas descritas pelo CFM para fundamentar essa resolução repousam nos Arts. 1° e 5° da Constituição Federal Brasileira, os quais estabelecem o princípio da dignidade da pessoa humana como um dos fundamentos do nosso país e proíbem a submissão de qualquer pessoa à tortura ou a tratamento desumano ou degradante. Em outras palavras, a resolução 1.805/2006 do CFM permitiu aos médicos a não implementação ou a suspensão de tratamentos com o intuito de preservar a dignidade das pessoas porque, em circunstâncias de terminalidade e na dependência dos seus valores e preferências de cuidados, medidas de suporte de vida poderiam corresponder a tratamentos desumanos e serem equivalentes a uma forma de tortura. Uma implicação extremamente relevante dessa resolução do CFM é que ela facilita a realização de testes terapêuticos envolvendo uma variedade de tratamentos, incluindo medidas de suporte de vida, algo que, muitas vezes, é essencial para se alcançar clareza sobre suas reais consequências e o impacto sobre a qualidade de vida do paciente. Se, uma vez que uma medida de suporte de vida houvesse sido iniciada, não fosse mais possível suspendê-la, o próprio conceito de teste terapêutico se tornaria irreal, e o processo de tomada de decisão seria dificultado imensamente.

Por exemplo, imaginemos a seguinte situação: um determinado paciente adulto de meia-idade com uma infecção respiratória grave por coronavírus da síndrome respiratória aguda grave 2 (SARS-CoV-2) ou outro vírus desenvolve insuficiência respiratória aguda, mas não autoriza a realização de VM, por ter medo de evoluir com "dependência de aparelhos para o resto da vida" ou "de morrer ligado a uma máquina na UTI". A possibilidade de ser assegurado pelo médico responsável por seus cuidados de que há uma chance concreta de, mediante a introdução da VM e a internação na UTI, ele conseguir sobreviver à fase aguda da infecção e se recuperar, bem como que, se a evolução de seu caso for desfavorável, a VM poderá ser suspensa, de modo que ele não morra ligado ao respirador na UTI, pode desempenhar papel decisivo na escolha do paciente por aceitar a realização de um *trial* envolvendo tanto a UTI como a VM.

É importante reconhecer que um passo fundamental para que questões relacionadas à suspensão ou não introdução de tratamentos de suporte de vida passa pelo uso de uma linguagem em que os conceitos-chave tenham sido definidos de forma clara. Trata-se, de fato, de um pré-requisito essencial para prevenir erros de comunicação e de tomada de decisão. Nesse sentido, é fundamental compreender que o conceito de "medidas de suporte de vida" corresponde a intervenções cuja ação busca manter o funcionamento de diferentes órgãos e sistemas fisiológicos com a finalidade de prolongar a vida, mas que, por si só, são incapazes de reverter a doença subjacente do indivíduo.^(32)^ Essas medidas incluem a ressuscitação cardiopulmonar, a VM, a terapia renal substitutiva, o uso de drogas vasoativas, a nutrição e hidratação por vias artificiais e o uso de antimicrobianos. Outros conceitos fundamentais a deliberações clínicas e bioéticas envolvendo essas questões são o de intervenções fúteis em senso estrito e de tratamentos potencialmente inapropriados.

De acordo com um importante documento de consenso publicado em 2015 de forma conjunta por cinco sociedades internacionais de terapia intensiva, devem ser consideradas como fúteis em senso estrito apenas as intervenções médicas em que não existe a menor possibilidade de que alcancem seu objetivo fisiológico (por exemplo: aumentar a saturação de oxigênio e restaurar a circulação espontânea).^(4)^ Exemplos de intervenções fúteis em senso estrito envolvem o uso de antimicrobianos contra germes sabidamente resistentes a eles e a realização de ressuscitação cardiopulmonar em pacientes cujo motivo da parada cardíaca se deve à disfunção múltipla de órgãos refratária ou a sangramento de grande volume e que se sabe de antemão impossível de reverter.^(25)^ Já os tratamentos potencialmente inapropriados foram definidos como aqueles que possuem alguma possibilidade de atingir seu objetivo fisiológico, mas cuja base ética é questionável.^(33)^ A distinção entre tratamentos fúteis em senso estrito e os potencialmente inapropriados é fundamental, porque os primeiros, em senso estrito, são sempre um equívoco do ponto de vista técnico e bioético e não devem ser implementados, ou mantidos, em hipótese alguma. Já os tratamentos potencialmente inapropriados necessitam de um processo de deliberação compartilhado e mais complexo, que passa pela identificação de valores culturais dos pacientes e seus familiares, do valor que esses indivíduos atribuem a determinados estados de funcionalidade e das probabilidades de sucesso das intervenções.^(5)^ Isso se deve ao fato de que o conceito de tratamentos potencialmente inapropriados, em contraposição ao da futilidade em senso estrito, não depende exclusivamente de um julgamento técnico, mas envolve valores morais que frequentemente variam entre pessoas distintas. Naturalmente, isso não quer dizer que a comunicação acerca de pedidos de pacientes ou familiares por tratamentos fúteis em senso estrito não exija um diálogo marcado pela sensibilidade e pela humildade cultural.^(34)^ A tabela 1S do Material Suplementar ilustra um exemplo de comunicação adequada envolvendo esse tipo de situação. As tabelas 1 e 2 descrevem exemplos de tratamentos fúteis em senso estrito e tratamentos potencialmente inapropriados, bem como os motivos que justificam sua classificação de tal forma.

**Tabela 1:** Exemplos de tratamentos fúteis em senso estrito e justificativa para determinação desse julgamento

Exemplos de tratamentos fúteis em senso estrito	Justificativa
Iniciar procedimentos de ressuscitação cardiopulmonar em paciente com parada cardíaca devido à hipotensão refratária por choque séptico, a despeito do melhor suporte circulatório possível	Nessas situações, os procedimentos de ressuscitação cardiopulmonar não serão capazes de solucionar o problema da hipotensão que causou a parada cardíaca, e o objetivo fisiológico de reestabelecer a circulação espontânea não é factível
Iniciar procedimentos de ressuscitação cardiopulmonar em paciente com parada cardíaca devido à hipóxia refratária a despeito do melhor suporte ventilatório possível	Nessas situações, os procedimentos de ressuscitação cardiopulmonar não serão capazes de solucionar o problema da hipóxia refratária que causou a parada cardíaca, e o objetivo fisiológico de reestabelecer a circulação espontânea não é factível
Prescrição de medicamento antifúngico para paciente com sepse estafilocócica sem evidência clínica ou laboratorial de infecção fúngica	Não há qualquer base fisiopatológica para justificar esse tratamento e nem possibilidade de ele alcançar o objetivo fisiológico de tratar a infecção do paciente
Procedimentos de ressuscitação cardiopulmonar em pacientes com *rigor* ou *livor mortis* na ausência de hipotermia grave como causa da parada cardíaca	Na ausência de hipotermia grave, *rigor* e *livor mortis* são sinais clínicos de morte irreversível e, em geral, denotam haver decorrido mais de 1 hora da interrupção da circulação espontânea, não havendo possibilidade de recuperá-la^(35,36)^
Manter procedimentos de ressuscitação cardiopulmonar por mais de 30 minutos para pacientes em assistolia ou atividade elétrica sem pulso refratárias, que já receberam manobras de ressuscitação de qualidade e cujas causas potencialmente reversíveis já foram abordadas dentro das possibilidades existentes no contexto de atendimento em que os pacientes se encontram	Na ausência de novos elementos que indiquem que os procedimentos de ressuscitação cardiopulmonar, que já foram extensivamente tentados e fracassaram até então em proporcionar o retorno à circulação espontânea, poderiam tornar-se bem-sucedidos, sua manutenção, por si só, não será capaz de alcançar o objetivo fisiológico do retorno à circulação espontânea

**Tabela 2:** Exemplos de tratamentos potencialmente inapropriados e justificativa para determinação desse julgamento

Exemplos de tratamentos potencialmente inapropriados	Justificativa
Utilização de alimentação por sonda para pacientes com demência em fase avançada	De um lado, há evidências que sugerem que a alimentação por via artificial em pacientes com demência em fase avançada não aumenta sua probabilidade de sobrevivência por mais tempo e é associada a uma maior chance de eventos adversos, como lesões por pressão e uso de contenção física, em comparação com a nutrição de conforto.^(7)^ De outro lado, o objetivo fisiológico de prover calorias e nutrientes pode ser alcançado por meio dessas intervenções. Adicionalmente, cabe considerar que indivíduos com diferentes perspectivas culturais podem possuir referenciais distintos para pesar os riscos e benefícios da alimentação por sonda
Implementação de ventilação mecânica para paciente com câncer metastático	A presença de câncer metastático, por si só, não impede que o objetivo fisiológico da ventilação mecânica (por exemplo: reduzir a hipoxemia ou hipercarbia) seja alcançado. Em certos contextos, a ventilação mecânica poderá aumentar o tempo de vida do paciente e viabilizar a realização de objetivos do paciente (por exemplo: vivenciar a formatura de um neto). Em outras circunstâncias, o contexto e a cultura do paciente poderão interpretar tal procedimento como um "prolongamento indevido da morte"
Uso de antibióticos para tratamento de pneumonia aspirativa recorrente em paciente acamado com sequela grave de acidente vascular cerebral	Com exceção de situações de germes extremamente multirresistentes para os quais não haveria opção de antimicrobiano disponível, de forma geral, existe a possibilidade de tais agentes alcançarem seu objetivo fisiológico de eliminar bactérias responsáveis pela infecção do paciente. A adequação ou não da decisão por continuar prescrevendo antibióticos para essas infecções deve levar em consideração em que medida o paciente gostaria de ser mantido vivo em seu estado de saúde atual ou consideraria tal estado como uma condição pior que a morte, algo intimamente relacionado a seus valores pessoais
Iniciar hemodiálise para pacientes em estado vegetativo crônico	O estado vegetativo crônico por si só não impede a hemodiálise de alcançar seu objetivo fisiológico de depurar o sangue do paciente de substâncias que usualmente seriam retiradas pela função dos rins, caso ela se encontrasse preservada. A questão central aqui envolve um julgamento de valor sobre a relação risco/custo/benefício de introdução de um procedimento como esse para pacientes com probabilidades mínimas de recuperação de consciência e/ou funcionalidade

Infelizmente, ainda são comuns os casos em que o desconhecimento ou a incompreensão dessas definições e de suas implicações para o processo de tomada de decisão na área da saúde levam a condutas inadequadas e eticamente reprováveis, tanto no sentido de manutenção, como de suspensão e não introdução de medidas de suporte de vida. O caso citado na epígrafe deste posicionamento retrata uma situação em que a equipe de saúde implementou e manteve medidas de suporte de vida para uma paciente com doenças graves e incuráveis, quando ela apenas desejava uma morte sem dor e, preferencialmente, em seu domicílio. Se a equipe houvesse reconhecido que a instalação de VM invasiva para aquela paciente era um tratamento potencialmente inapropriado, ao tomar conhecimento dos valores claramente expressos pela paciente e por sua família, teria a reconhecido como definitivamente inapropriada, e tal tratamento sequer teria sido iniciado.

Outro caso publicado recentemente no *New England Journal of Medicine* retratou a situação de um homem de meia-idade com dependência funcional decorrente de uma deficiência neurológica associada à epilepsia de difícil controle, a quedas frequentes e a traumatismo craniano recorrente.^(37)^ O paciente fora hospitalizado devido a uma pneumonia aspirativa e, apesar de ter se recuperado da pneumonia mediante antibioticoterapia, mantinha quadro de disfagia com alto risco de aspiração. A família do paciente, que incluía uma fonoaudióloga, acreditava que uma gastrostomia seria invasiva demais e que existia uma chance razoável de o paciente recobrar a capacidade de se alimentar pela boca, caso ele fosse alimentado temporariamente através de uma sonda nasogástrica, inclusive porque episódios semelhantes já tinham ocorrido no passado, e ele se recuperara, contra a expectativa de muitos. De acordo com o ponto de vista da equipe de saúde, uma sonda nasogástrica seria uma fonte de sofrimento para o paciente. Por outro lado, sua família não compartilhava desse julgamento, inclusive porque, no passado, o paciente já havia utilizado esse tipo de sonda por um certo período e demonstrara tolerá-la bem. A despeito dos pedidos de sua família por um período de teste de alimentação por sonda nasogástrica, o paciente recebeu alta hospitalar com recomendação de dieta geral por via oral, desenvolveu novo episódio de pneumonia, e, sob pressão da equipe de saúde, sua família consentiu que ele fosse encaminhado para um *hospice*, onde faleceu após poucos dias. O relato desse caso possui em comum com o anterior o fato de que, em ambos, a equipe de saúde também não foi capaz de escutar e considerar os valores do paciente e de sua família durante o processo de tomada de decisão. A diferença é que, no segundo caso, uma medida de suporte artificial de vida - a passagem da sonda -, em um primeiro momento, deveria ter sido reconhecida como potencialmente inapropriada e, mediante a escuta e a ponderação dos valores do binômio paciente/família, tornar-se-ia provavelmente adequada.

Adicionalmente, é essencial reconhecer que a suspensão e a não implementação de intervenções de suporte de vida no contexto de uma doença terminal diferem do conceito de eutanásia. A *International Association for Hospice and Palliative Care* (IAHPC) e a *European Association for Palliative Care* (EAPC) publicaram posicionamentos acerca da questão da eutanásia,^(38,39)^ definindo-a como a ação de um médico em administrar medicamentos a um paciente com o intuito de causar sua morte com base em uma solicitação voluntária do paciente feita na vigência de plena capacidade de decisão. O documento da EAPC defende ainda que o termo "eutanásia passiva" é inapropriado, por representar uma contradição intrínseca, na medida em que a eutanásia, conforme já definida, corresponderia sempre a uma ação, sendo, portanto, essencialmente ativa.^(39)^ Merece destaque que ambas as associações se posicionaram veementemente contra a eutanásia e afirmaram que ela não integra a visão filosófica contida na definição de cuidados paliativos^(40,41)^ e que, portanto, não deveria integrar a prática desses cuidados. Tanto a IAHPC como a EAPC foram categóricas ao afirmarem que a prática de suspender ou não iniciar medidas de suporte de vida para pacientes em fase terminal de doenças graves e incuráveis não corresponde à eutanásia. Um dos argumentos centrais que diferencia a eutanásia da prática de suspensão/não implementação de intervenções de suporte de vida repousa no fato de que, na primeira situação, os medicamentos administrados são a causa efetiva da morte, enquanto, no segundo caso, a morte é causada pela doença subjacente e apenas deixa de ser prolongada por meio da suspensão ou da não implementação dos tratamentos.

A legalidade da suspensão e da não implementação de medidas de suporte de vida já é uma realidade em vários países, como Reino Unido, Holanda, Austrália, Colômbia, Taiwan e Estados Unidos.^(32)^ Naturalmente, a questão da regulamentação ou não dessas práticas em cada país é influenciada por sua cultura, seus valores, sua economia e a orientação religiosa.^(42)^ Adicionalmente, é importante considerar que esses aspectos podem se modificar ao longo do tempo e da história dos países, de tal modo que a suspensão e a não implementação de tratamentos de suporte de vida, as quais um dia foram proibidas, muitas vezes passam a ser aceitas em momentos futuros.

Percebe-se que, mesmo em localidades onde as práticas da suspensão e da não implementação de medidas de suporte de vida são regulamentadas por lei, o conhecimento dos profissionais de saúde ainda é escasso a esse respeito, levando até mesmo à adoção de tratamentos fúteis em sentido estrito, diante de condições críticas, em pacientes no fim da vida.^(32)^

É importante ressaltar que a prática dos cuidados paliativos vai muito além da mera questão da retirada ou da não introdução de medidas de suporte de vida. O cuidado paliativo visa prevenir e aliviar o sofrimento associado a doenças graves e ameaçadoras da vida e possui, sim, o papel de ajustar e, muitas vezes, retirar intervenções e tratamentos que estejam causando ou prolongando sofrimento em uma situação de adoecimento irreversível. Devemos ser cuidadosos, no entanto, e nos certificar de que estamos definindo sofrimento de acordo com a percepção e os valores do paciente, não dos nossos próprios conceitos. Infelizmente, o termo "distanásia", que corresponde ao prolongamento exagerado da morte,^(43)^ tem sido usado de forma indevida, tomando por base apenas a perspectiva de valores de profissionais de saúde para qualificar o "exagero" como toda situação em que os mesmos considerariam que "não vale a pena viver", sem uma exploração adequada dos valores do paciente sobre sofrimento e o que vale a pena viver do seu ponto de vista.

Infelizmente, em nosso país, temos um défice importante de profissionais e equipes especializadas em cuidados paliativos, sendo que 55% dos serviços existentes encontram-se concentrados na Região Sudeste.^(44)^ Considerando esse défice, restringir a prática do ajuste de suporte terapêutico, incluindo a suspensão e a não introdução de tratamentos de suporte de vida, a locais com acesso a especialistas em cuidados paliativos teria o impacto de limitar o acesso de pacientes à possibilidade de ter seus valores respeitados e evitar um processo de morte prolongada artificialmente contra seus valores pessoais. Avaliamos, portanto, que, apesar do ideal ser contar com uma equipe especializada^(45)^ (presencialmente ou através de telemedicina), o ajuste de plano terapêutico faz parte das habilidades primárias de cuidado paliativo, esperadas de todos os médicos, conforme recentemente reconhecido pela atualização das diretrizes curriculares nacionais para a graduação em medicina no Brasil.^(46)^

Finalmente, o complexo processo de decisão compartilhada, envolvendo empregar ou não medidas de suporte de vida, requer um elevado grau de autovigilância, não apenas para evitarmos impor, ainda que de forma velada, nossas perspectivas culturais,^(5)^ mas também para que não lancemos mão de linguagem que induza percepções equivocadas em pacientes e familiares.^(47)^ Por exemplo, o mal uso dos verbos "precisar" e "necessitar" ao descrever certa opção terapêutica (por exemplo: "se os níveis de oxigênio continuarem caindo, ele vai *precisar* ser intubado e colocado em VM") transmite a falsa impressão de que essa é a única alternativa razoável. No exemplo, seria mais apropriado usar linguagem do tipo "se os níveis de oxigênio continuarem caindo, nossas opções são… e envolvem tais vantagens e desvantagens, riscos e benefícios… e podem fazer sentido para algumas pessoas e não para outras, dependendo de seus valores e preferências".

Lamentavelmente, não são raras as situações em que pacientes e familiares são confrontados por profissionais de saúde com a falsa dualidade de, de um lado, prolongar a vida aumentando o sofrimento da pessoa ou, de outro lado, deixar de instituir medidas de suporte de vida como estratégia para evitar o sofrimento. Esse tipo de abordagem é inapropriado, do ponto de vista técnico e bioético, porque, independentemente da preferência por adotar ou não medidas para prolongar a vida de uma pessoa dentro dos limites existentes, os profissionais de saúde têm sempre o compromisso de fazer o melhor possível para reduzir o sofrimento de seus pacientes. Em última instância, o sofrimento grave e refratário pode ser aliviado mediante o uso de sedação paliativa até a inconsciência, independentemente da decisão pela utilização ou não de medidas de suporte de vida.^(48,49)^ Naturalmente, a opção pela sedação paliativa até a inconsciência corresponde a uma estratégia de último recurso para alívio de sintomas desconfortáveis que receberam avaliação e tratamentos apropriados, todavia sem sucesso.^(50)^

### Posicionamento da Academia Nacional de Cuidados Paliativos

Tendo em vista as considerações apresentadas no preâmbulo, o Comitê de Bioética da Academia Nacional de Cuidados Paliativos emite os seguintes posicionamentos:

1. Do ponto de vista da bioética, não existe distinção efetiva entre a retirada e a não implementação de intervenções de suporte de vida. Decisões acerca da retirada ou não implementação desse tipo de tratamento devem sempre seguir os princípios da tomada de decisão compartilhada e requerem a concordância do paciente ou de seu representante legal, para que possam ser realizadas.
2. Não é ético iniciar ou manter medidas de suporte de vida em contextos em que estas estejam em desacordo com os valores e objetivos de cuidado definidos em conjunto com pacientes e seus familiares, por exemplo, em situações compreendidas por eles como piores que a própria morte.
3. Tratamentos fúteis em senso estrito são aqueles que não possuem qualquer possibilidade de alcançar sequer seu objetivo fisiológico. Por definição, não são capazes de prolongar a vida, pois são simplesmente ineficazes. Portanto, tratamentos fúteis em senso estrito não devem ser iniciados ou mantidos nem mesmo a pedido do paciente ou de seus representantes.
4. Se a manutenção de determinado tratamento é capaz de prolongar a vida de um paciente, mas existe um questionamento sobre sua base ética, tal tratamento não pode ser considerado fútil em senso estrito e corresponde, na verdade, a um tratamento potencialmente inapropriado. A suspensão ou não implementação de tratamentos de suporte de vida potencialmente inapropriados requer, obrigatoriamente, a pactuação de objetivos terapêuticos, levando em consideração os valores e as perspectivas do paciente.
5. É importante reconhecer que profissionais de saúde, pacientes e seus entes queridos podem perceber a retirada e a não implementação de intervenções de suporte de vida como situações com pesos e impactos distintos. Nesses casos, devem ser utilizadas abordagens dialógicas capazes de, com sensibilidade e empatia, trazer clareza a todos os envolvidos de que a questão central a ser definida diz respeito à adequação de qualquer intervenção, independentemente de ela já ter sido iniciada ou não, aos objetivos de cuidados pactuados entre profissionais de saúde, pacientes e seus familiares.
6. É essencial reconhecer que a objeção de consciência por parte dos profissionais de saúde possui limites, os quais envolvem situações em que ela causa sofrimento, danos ou a morte ao paciente ou em que haja ausência de outro profissional de saúde para assumir a responsabilidade quanto à realização, à não implementação ou mesmo à suspensão de determinado procedimento, em tempo hábil.
7. Ao discutir com pacientes/familiares a questão da adequação ou não de suspender ou não implementar medidas de suporte de vida, os profissionais de saúde devem evitar usar linguagem que induza pacientes/familiares a acreditarem que apenas uma das opções disponíveis relacionadas à utilização ou não de medidas de suporte de vida possibilitará o alívio do sofrimento. O compromisso ético pelo alívio do sofrimento não deve ser influenciado de forma alguma pela decisão por empregar ou não intervenções de suporte de vida.
